# Mitogenomics of *Perumytilus purpuratus* (Bivalvia: Mytilidae) and its implications for doubly uniparental inheritance of mitochondria

**DOI:** 10.7717/peerj.5593

**Published:** 2018-09-18

**Authors:** Beata Śmietanka, Marek Lubośny, Aleksandra Przyłucka, Karin Gérard, Artur Burzyński

**Affiliations:** 1 Department of Genetics and Marine Biotechnology, Institute of Oceanology Polish Academy of Sciences, Sopot, Poland; 2 Centro de Investigacion Gaia-Antartica, Departamento de Recursos Naturales, Universidad de Magallanes, Punta Arenas, Chile; 3 Laboratorio de Ecosistemas Marinos Antárticos y Subantárticos, Universidad de Magallanes, Punta Arenas, Chile

**Keywords:** DUI, mtDNA

## Abstract

Animal mitochondria are usually inherited through the maternal lineage. The exceptional system allowing fathers to transmit their mitochondria to the offspring exists in some bivalves. Its taxonomic spread is poorly understood and new mitogenomic data are needed to fill the gap. Here, we present for the first time the two divergent mitogenomes from Chilean mussel *Perumytilus purpuratus.* The existence of these sex-specific mitogenomes confirms that this species has the doubly uniparental inheritance (DUI) of mitochondria. The genetic distance between the two mitochondrial lineages in *P. purpuratus* is not only much bigger than in the *Mytilus edulis* species complex but also greater than the distance observed in *Musculista senhousia*, the only other DUI-positive member of the Mytilidae family for which both complete mitochondrial genomes were published to date. One additional, long ORF (open reading frame) is present exclusively in the maternal mitogenome of *P. purpuratus*. This ORF evolves under purifying selection, and will likely be a target for future DUI research.

## Introduction

The marine mussel *Perumytilus purpuratus* (Lamarck, 1819), an endemic species belonging to the Mytilidae family, forms extensive banks in the mid-intertidal rocky shores of the south-west coasts of South America (Guinez & Castilla, 1999). This species is known from intense intraspecific competition within multi-stratified matrices of individuals (Guiñez, 2005; Acevedo, Orellana & Guiñez, 2010). The distribution of *P. purpuratus* ranges along Pacific coast from 2°S in Ecuador to 56°S in Chile, and extends around Cape Horn up to 41°S in Argentinean Patagonia (Zagal, 2001). In contrast to most marine mussels with a long-lasting planktotrophic larval stage and high dispersal capability, *P. purpuratus* has a lecithotrophic development, with a larval stage lasting only 14–18 days under laboratory conditions (Garrido, 1996), leading to limited potential for dispersal. This, together with Pleistocene separation of Atlantic and Pacific populations, led to the relatively deep and well-defined contemporary split of the species into two populations: south-eastern and northern, with a clear genetic discontinuity close to the 40° S latitude in the Pacific (Trovant et al., 2015; Guiñez et al., 2016). It has been suggested, based on the anatomy of sperm, that these two populations may even represent two separate species (Briones et al., 2012).

In most animals, mitochondrial DNA (mtDNA) undergoes strictly maternal inheritance (SMI) (Avise, 1986; Birky, 1995; Birky, 2001). However, in some bivalve species an alternative way termed doubly uniparental inheritance (DUI) has been discovered (Skibinski, Gallagher & Beynon, 1994b; Zouros et al., 1994). Under DUI, females transmit their mtDNA (F genome) to the offspring the same way as under SMI. However, males have an additional mtDNA (M genome) which is transmitted preferentially to sons. As a result, females are homoplasmic but males are heteroplasmic as they inherit mtDNA from both parents. The phenomenon was first observed in blue mussels, *Mytilus edulis* but has been confirmed in several bivalvian families (Zouros, 2013). The two mitochondrial lineages have a variable level of genetic divergence, up to 51% of uncorrected amino acid p-distance in freshwater mussels (Unionidae) (Doucet-Beaupré et al., 2010). It has been suggested that the M genome evolves under relaxed selection, and faster than the F genome (Skibinski, Gallagher & Beynon, 1994a; Rawson & Hilbish, 1995), although it is unclear what causes these phenomena (Skibinski et al., 2017). Several unusual features of the mitogenomes present in DUI species have been reported. These include cases of mtDNA recombination (Ladoukakis & Zouros, 2001; Burzyński et al., 2003; Burzyński & Śmietanka, 2009) as well as the presence of atypical genes, either in the form of sex-specific gene extensions (Chapman et al., 2008) or complete sex-specific open reading frames (ORFs) (Breton et al., 2009; Milani et al., 2013). It has been postulated that these features may play a role in the maintenance of DUI and in sex determination, in the groups of DUI bivalves in which they were discovered (Breton et al., 2011b; Minoiu, Burzyński & Breton, 2016; Guerra et al., 2017). However, both relatively poor taxonomic sampling of species with DUI and the variety of mitogenomic features found in the different groups of bivalves make any attempts at drawing general conclusions very difficult. It is therefore important to extend our knowledge of the mitogenomics of DUI species, particularly species from families already known for this phenomenon.

It has been suggested, that DUI may be present in *P. purpuratus* (Vargas et al., 2015). However, the suggestion was based on sequencing of short fragments of *16S* and *cox1* mitochondrial genes from the northern population only. To verify this suggestion, and check if the mitogenomes of this species also contain unusual features, possibly related to DUI, we sequenced complete mitogenomes from both: northern and south-eastern *P. purpuratus* populations.

## Methods

Mussels were sampled at the Chilean coasts of Pacific Ocean from two locations: near Las Cruces (33°29′09.60″S, 71°38′40.97″W) representing the northern population in January 2014, and London Island in the Brecknock Passage (54°42′9.75″S, 71°54′50.06″W) representing the south-eastern population in October 2015. Animals were sexed by microscopic examination of mature gonads. Gonadal tissue samples were kept in 70% ethanol until use. One male was selected from each sample for full mitogenome sequencing. The material used in this study is kept in repository at IO PAN (Institute of Oceanology Polish Academy of Sciences) under the following voucher numbers: LCP1 and BRP4 for the northern and south-eastern samples, respectively.

To verify the presence of DUI the following procedure was used for LCP1 sample. RNA was extracted and sequenced by RNA-Seq (Illumina MiSeq platform, Macrogen, South Korea) to verify the presence of two sets of transcripts, as described previously (Sańko & Burzyński, 2014; Lubośny et al., 2017; Soroka et al., 2018). The resulting raw reads are available in SRA database under accession number SRR7504388. After positive verification of the presence of two sets of transcripts, DNA was extracted from the same sample, using established methodology (Hoarau et al., 2002). Based on mitotranscript sequences two pairs of Long Range Polymerase Chain Reaction (LR-PCR) primers (Table S1) were designed using primer3 software (Untergasser et al., 2012) and used to amplify the complete M and F mitogenomes in two overlapping fragments. These fragments were then sent to Macrogen (South Korea) for next generation sequencing (NGS) on Illumina MiSeq platform. Two separate libraries were prepared: one for amplicons obtained from the F mitogenome and one for amplicons obtained from the M mitogenome. The raw results are available in SRA database under the following accession numbers: SRR7504346 (F) and SRR7504347 (M). Raw reads from both libraries were assembled in CLC Genomics Workbench 9.0 (Qiagene, Vedbæk, Denmark) into single contigs containing complete mitochondrial genomes. There were enough reads to achieve average mitogenome coverage of more than 50, 000 ×, making the result of the assembly very reliable.

The positive results obtained for the northern population along with the falling prices for NGS services, prompted us to simplify the methodology, and so we did not do the verification of mitotranscripts for the south-eastern population. Since the genetic divergence between the populations was known to be relatively high (Trovant et al., 2015), it was unlikely that the LR primers designed for LCP1 sample would work for BRP4. Therefore, it was decided to attempt direct deep sequencing of genomic DNA isolated from mature male gonads. It was expected that this would produce enough mitogenomic reads to assembly both M and F mitogenomes, at least partially. DNA was isolated from sample BRP4 and send to Macrogen (South Korea) for NGS sequencing. A single Illumina Ten-X NGS run was performed. In this case, the reads were assembled in NOVOplasty (Dierckxsens, MarDulyn & Smits, 2017). Previously assembled M and F mitogenomes from the northern population were used as seeds, but the seed sequences did not contribute to the final assembly. Both mitogenomes were recovered in two separate runs, as single, circular contigs. Apparently there was enough mitochondrial reads to recover the complete mitogenomic sequences. These raw reads are available in SRA database under accession number SRR7504397. The average coverage of the final assembly was 40 × for the F mitogenome and 160 × for the M mitogenome and there were no gaps in coverage requiring further attention.

Bowtie (Langmead & Salzberg, 2012) was used to map the reads back to the assembled contigs and to verify the coverage. The contigs were annotated as described previously (Zbawicka, Burzyński & Wenne, 2007), using CRITICA for identification of coding sequences (Badger & Olsen, 1999), GLIMMER to assess the span of the reading frames (Delcher et al., 1999), BLAST to confirm the identity of the genes (Altschul et al., 1990; Zhang & Madden, 1997), Wise2 to predict the location of typical mitochondrial protein coding genes (Birney, Clamp & Durbin, 2004), HMMER to generate hmm profiles and to identify rRNA genes (Wheeler & Eddy, 2013), ARWEN to identify tRNA genes (Laslett & Canbäck, 2008). Annotated mitogenomic sequences have been deposited in GenBank under the following accession numbers: MH330330 (LCP1, M), MH330331 (BRP4, M), MH330332 (LCP1, F), MH330333 (BRP4, F).

MEGA7 (Kumar, Stecher & Tamura, 2016) was used to align sequences, calculate genetic distances and reconstruct phylogeny of *trn* genes. The evolutionary history was inferred by using the Maximum Likelihood (ML) method based on the General Time Reversible (GTR) model (Nei & Kumar, 2000). Stability of tree topology was tested by bootstrap procedure with 500 replicates (Felsenstein, 1985). Sliding window analysis of genetic diversity was done in DnaSP (Librado & Rozas, 2009). Circular maps of male and female genomes and nucleotide composition analyses were produced in CGView (Stothard & Wishart, 2005). Predictions of transmembrane domains were done in CLC Genomics Workbench version 9.0 (QIAGENE).

To evaluate selective pressure acting on the mitogenomes, the representative fragments of the genomes were chosen. Specific primers were designed (Table S1), the fragments of *nd5* gene as well as the complete f-ORF gene were amplified and sequenced from a number of individuals (34 for *nd5* and 17 for f-ORF). The PCR amplification protocol was as follows: initial denaturation at 94 °C for 2 min, followed by 30 cycles of denaturation at 93.5 °C 30 s, annealing at 58 °C for 30 s, and extension at 72 °C for 1 min 40 s. The final extension lasted 5 min. Sequences were obtained directly from PCR products, using BigDye terminator chemistry (ABI) in Macrogen (South Korea). Raw sequences were trimmed and assembled using the Staden package (Bonfield, Smith & Staden, 1995), and deposited in GenBank under the following accession numbers: MH645667–MH645717. The sequences were aligned in MEGA7, following which the number of nonsynonymous substitutions per nonsynonymous site (dN) as well as the number of synonymous substitutions per synonymous site (dS) were calculated. Standard error estimates (SE) were obtained by a bootstrap procedure (100 replicates). Analyses were conducted using the Nei-Gojobori model (Nei & Gojobori, 1986). All positions containing gaps and missing data were eliminated.

**Figure 1:**
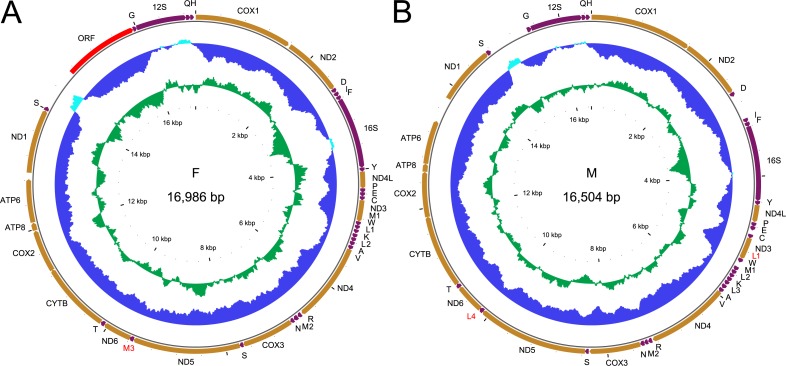
Genetic map of the two mitogenomes of *Perumytilus purpuratus*. F mitogenome (A); M mitogenome (B)*.* All protein coding genes (in orange) are labeled by the names of the encoded proteins, two *rRNA* genes (in purple) are labeled 16S and 12S for large and small subunit, respectively. The *trn* genes (also in purple) are labeled by the one letter code of the respective amino acid. Additional genes discussed in text are labeled in red. The direction of transcription is indicated by the position and direction of the arrows, all genes are transcribed clockwise. The two inner circles represent local compositional bias, calculated in a 200 bp long window with 25 bp steps. The light blue parts of the first circle represent positive AT skew while the dark blue parts of this circle represent negative AT skews. The inner, green circle represents local GC content, relative to the average for each genome.

## Results

**Figure 2:**
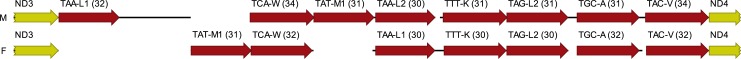
Alignment of a fragment of F and M mitogenomes located between *nd3* and *nd4*. This part of mitogenomes contain several *trn* genes. They are labeled with their anticodon and amino acid one letter code. Additionally, *trn* genes of the same amino acid specificity are numbered consecutively, along the genome (as in Fig. 1). All the *trn* genes presented here were predicted with high confidence by ARWEN. The bitscore for each gene prediction is shown in bracket. The L1 gene of the F mitogenome is aligned with the L2 gene of the M mitogenome while the positions of M1 and W are switched.

**Figure 3:**
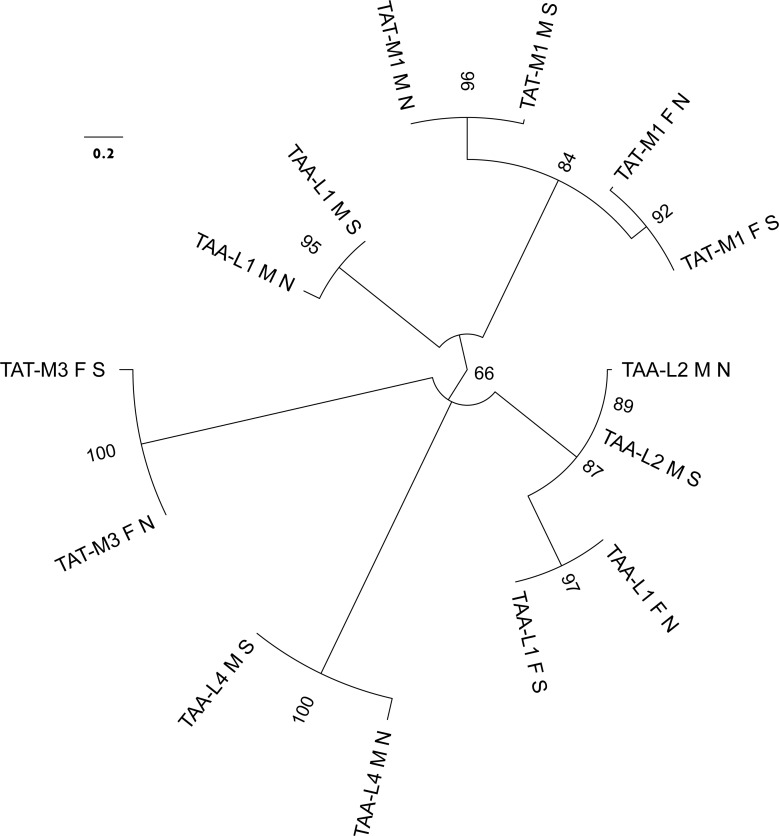
Molecular phylogenetic analysis by Maximum Likelihood method of the relatioships between *trn* genes with TAT and TAA anticodons. The evolutionary history was inferred by using the ML method based on the General Time Reversible model. The tree with the highest log likelihood (−420.5468) is shown. The percentage of trees in which the associated taxa clustered together is shown next to the branches. Initial tree(s) for the heuristic search were obtained automatically by applying Neighbor-Join and BioNJ algorithms to a matrix of pairwise distances estimated using the Maximum Composite Likelihood (MCL) approach, and then selecting the topology with superior log likelihood value. A discrete Gamma distribution was used to model evolutionary rate differences among sites (4 categories (+G, parameter = 7.2811)). The tree is drawn to scale, with branch lengths measured in the number of substitutions per site. The analysis involved 14 nucleotide sequences. All positions containing gaps and missing data were eliminated. There were a total of 49 positions in the final data set. The leaves are labeled by the anticodon and amino acid specificity with consecutive numbers (as in Fig. 1), by the lineage (F or M) and by the population (N for northern, S for south-eastern).

Complete F mitogenomes of *P. purpuratus* sampled from northern and south-eastern populations are similar in length (16,963 bp and 16,986 bp respectively) and have the same gene content and order. The M mitogenomes are shorter (16,464 bp and 16,504 bp respectively) but also do not differ in gene content and order. The genetic maps of M and F mitogenomes from BRP4 are presented in Fig. 1. The maps of mitogenomes from LCP1 are identical. Both M and F mitogenomes include a set of genes typical for a metazoan mitogenome (Avise, 1986): 13 parts of OXPHOS (oxidative phosphorylation) machinery, two subunits of mitochondrial ribosomal RNA and 22 tRNAs needed to express them. However, more *trn* genes were identified by ARWEN, all with TAA or TAT anticodons. Additional *trnMET* with TAT anticodon was annotated in all mitogenomes (Fig. 2), and the F mitogenomes contain two such genes (Fig. 1), for the total of three *trnMET* genes and 24 *trn* genes. The M genomes contain two additional *trnLEU* with TAA anticodons, bringing the total number of *trn* genes to 25. All these genes were annotated by ARWEN with bitscores much higher than the minimum 20 expected for true *trn* genes (Fig. 2). There is also a small difference in the order of *trn* genes within the block located between *nd3* and *nd4* (Fig. 2): the order of *trnMET1* and *trnTRP* is reversed, suggesting that a duplication-random-loss event may have been involved in shaping this pattern. An attempt has been made to reconstruct the phylogenetic relationships among these genes (Fig. 3). The reconstructed phylogeny is not fully resolved, but *trn* genes do not form monophyletic, anticodons-specific clades.

**Figure 4:**
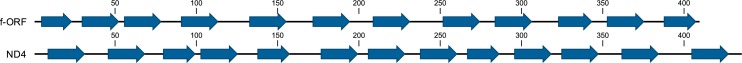
Prediction of transmembrane helices in ND4 and putative f-ORF proteins. The amino acid positions of the predicted helices is shown along each sequence.

The most striking difference between the M and F mitogenomes of *P. purpuratus* is the existence of one additional, 1,227 bp long f-ORF in the F mitogenome, directly adjacent to the longest non-coding region (14718..15944 in BRP4, 14693..15919 in LCP1). This f-ORF does not have any significant BLAST hits in NCBI *nr* database. However, limited similarity to *P. purpuratus* ND4 (28% identity in BLAST over 106 bp long local alignment) exists. Moreover, the predicted protein contains a set of 13 transmembrane domains, a pattern similar to the one observed in this ND4 protein (Fig. 4).

**Figure 5:**
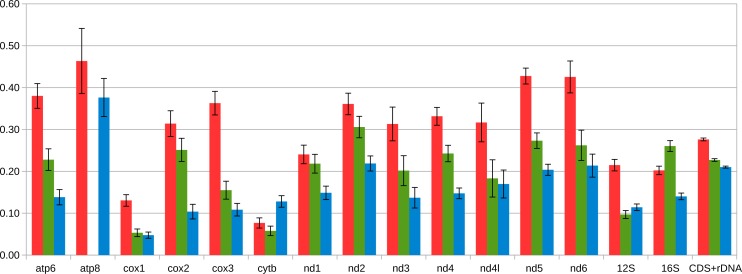
Average distance between coding part of M and F mitogenomes in Mytilidae. The following sets of mitogenomes are compared: the three species of the *Mytilus edulis* complex in which the speciation predated the diversification of M and F lineages (three pairs of mitogenomes, in blue), *Musculista senhousia*, having higher M-F divergence than *Mytilus* (one pair of mitogenomes, in green), and *Perumytilus purpuratus* (two pairs of mitogenomes, in red). Each protein coding and rRNA sequence was extracted and aligned separately for each genus. Then the average between-group amino acid p-distance was calculated in MEGA7. Standard error estimates (shown in figure) were calculated by bootrstrap procedure (500 replicates). Distances for rRNA genes as well as for concatenated alignment of all genes considered were calculated as nucleotide p-distances (but are shown on the same scale). The *atp8* gene has not been annotated in *Musculista*.

**Figure 6:**
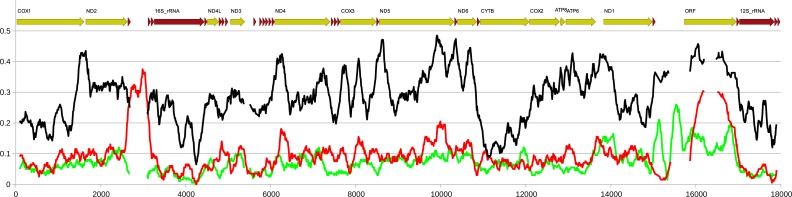
Comparison of genetic diversity at interpopulation and interlineage level in *Perumytilus purpuratus*. Nucleotide diversity calculated in a sliding window of 200 bp in 10 bp steps along the alignment of the four mitogenomes. Red line indicates nucleotide diversity within the group of two M genomes, green line indicates the diversity within the group of two F genomes while the black line indicates the distance between the two groups, expressed as Dxy. The calculations were done in DnaSP. The approximate positions of the genes in the alignments is shown above the plot.

Except for the f-ORF and the mentioned *trn* genes all four mitogenomes are co-linear, allowing unambiguous alignment and assessment of genetic distance between them. The average uncorrected nucleotide p-distance between F mitogenomes sampled from different populations is 0.065 whereas the distance between the two M mitogenomes is 0.085. The average distance between M and F mitogenomes is much greater (0.28). However, when individual genes are compared (Table 1, Tables S2, S3), they show marked differences. Comparative analysis involving representatives of the two other genera from Mytilidae family was made to better illustrate these differences (Fig. 5). It shows that while *cox1* (usually the least diversified protein coding gene), it is also among the most conserved genes in *P. purpuratus* M-F comparison, the least divergence is accumulated in the *cytb* gene. The most diversified are members of the dehydrogenase complex: *nd2-6*. Sliding window approach (Fig. 6) confirmed that notion, but also showed that substitutions at intra-lineage level frequently follow the same pattern as in the interlineage (M-F) context.

**Table 1:** Length, base composition (%), and distances (K) between M and F genes of *Perumytilus purpuratus*. Gene lengths for south-eastern (S) and northern (N) populations representatives are shown. All estimates except for f-ORF, are averages between two intra-population (M-F) comparisons. Individual comparisons, separately for each population are presented in Tables S2 and S3. The data for f-ORF (the last line of the table) are for comparison of the sequence from two sequenced F mitogenomes (LCP1 and BRP4). The indices were calculated by counting the number of synonymous substitutions per synonymous site (Ks) and the number of nonsynonymous substitutions per nonsynonymous site (Ka). Modified Nei-Gojobori model was used, and for calculating genetic distances (K), Tamura 3- parameter model was applied.

Gene		Length (bp)		Base composition (%)				M-F distance		
Name	Lineage	S	N	T	C	A	G	K	Ks	Ka
*16S*	F	1,161	1,161	39.7	10.2	32.4	17.5	0.25		
	M	1,187	1,179	40.8	9.9	31.0	18.5			
*12S*	F	818	819	33.7	11.6	34.0	20.8	0.25		
	M	815	819	35.1	11.7	33.2	20.4			
*atp6*	F	702	702	45.1	10.3	24.8	19.9	0.47	0.68	0.38
	M	690	687	46.5	11.5	20.2	21.9			
*atp8*	F	123	123	49.2	4.9	24.4	21.6	0.57	0.61	0.45
	M	123	123	51.2	5.7	14.2	28.9			
*cox1*	F	1,589	1,587	41.9	13.5	23.9	20.8	0.14	0.83	0.11
	M	1,605	1,611	41.2	14.2	22.7	22.0			
*cox2*	F	690	690	39.8	12.1	26.8	21.4	0.33	0.77	0.31
	M	708	711	38.6	13.5	25.4	22.6			
*cox3*	F	846	846	43.4	11.2	22.9	22.6	0.42	0.67	0.32
	M	804	804	45.0	11.4	19.8	23.9			
*cytb*	F	1,155	1,155	43.5	13.2	25.4	17.9	0.08	0.53	0.05
	M	1,155	1,155	44.4	12.6	23.4	19.7			
*nd1*	F	1,044	1,044	43.7	10.7	23.6	22.1	0.29	0.80	0.18
	M	937	937	44.8	11.0	19.5	24.7			
*nd2*	F	984	984	44.3	11.8	22.3	21.8	0.45	0.79	0.32
	M	987	984	46.3	9.7	21.4	22.7			
*nd3*	F	345	345	44.1	11.2	21.3	22.3	0.37	0.80	0.25
	M	345	345	49.6	10.6	17.9	23.2			
*nd4*	F	1,302	1,302	43.5	10.5	24.2	21.9	0.58	0.62	0.35
	M	1,302	1,302	45.9	9.7	20.6	23.9			
*nd4L*	F	270	270	40.9	10.6	23.4	25.2	0.31	0.73	0.26
	M	270	270	42.4	9.1	20.6	28.0			
*nd5*	F	1,743	1,743	43.2	10.8	24.7	21.5	0.47	0.73	0.41
	M	1,749	1,758	42.6	12.1	22.9	22.6			
*nd6*	F	462	462	46.9	8.5	25.0	20.8	0.59	0.78	0.44
	M	462	462	47.5	10.4	19.5	22.7			
f-ORF	F	1,224	1,224	50.05	10.2	23.3	16.45	0.15	0.26	0.11

To comparatively assess selective pressure acting on putative f-ORF sequence, small samples of sequences were collected from the quickly evolving *nd5* gene from M and F mitogenomes as well as from f-ORF (Table 2). Both intrapopulation and interpopulation comparisons suggest that the purifying selection acting on M mitogenomes is weaker than the one acting on the F mitogenomes, because at *nd5* the dN/dS ratio is lower for F than for M data. This is true, even in the context of apparently higher intrapopulation genetic polymorphism observed in the F data. However, the selective pressure is even weaker for the f-ORF. Nevertheless, the pattern of polymorphisms for this gene is also consistent with purifying selection (Table 2, dN/dS < 1).

**Table 2:** Estimates of average codon-based evolutionary divergence within two populations of *Perumytilus purpuratus*. Two mitogenomic regions were analyzed: 950 bp long fragments of *nd5* from both M and F mitogenomes and a complete, 1,227 bp long f-ORF from the F mitogenome. Sequences were sampled from the northern (N) and south-eastern (S) populations. The nucleotide diversity, expressed as the numbers of substitutions per site (*d*) was calculated for all groups. The number of nonsynonymous substitutions per nonsynonymous site (*dN*) as well as the number of synonymous substitutions per synonymous site (*dS*) from averaging over all sequence pairs in each group are shown. These analyses were conducted using the Nei-Gojobori model for scoring synonymous and non-synonymous sites. There were approximately 715 non-synonymous sites in the ND5 data sets and 954 non-synonymous sites in the f-ORF data set. The number of involved nucleotide sequences (*n*) is shown in the last column. All positions containing gaps and missing data were eliminated. Standard error estimates were obtained by a bootstrap procedure (100 replicates) and used in rounding up the numbers accordingly.

Gene-lineage	Sample	*d*	*dN*	*dS*	*dN/dS*	*n*
ND5 M	S	0.0043	0.0007	0.0162	0.04	4
	N	0.0022	0.0004	0.0080	0.04	15
	S+N	0.0331	0.0126	0.1233	0.10	19
ND5 F	S	0.0335	0.0054	0.1477	0.04	6
	N	0.0109	0.0009	0.0440	0.02	9
	S+N	0.0421	0.0061	0.1914	0.03	15
f-ORF F	S	0.0169	0.0072	0.0544	0.13	6
	N	0.0172	0.0088	0.0485	0.18	11
	S+N	0.0768	0.0545	0.2090	0.26	17

## Discussion

The presence of two divergent mitogenomes following the gender-specific tissue distribution fully confirm the suggestions (Vargas et al., 2015) that *P. purpuratus* has the DUI of mitochondria. The genetic distance between M and F mitochondrial lineages in *P.* purp*uratus* is much higher than in *Mytilus edulis* species complex (Breton, 2005; Zbawicka, Burzyński & Wenne, 2007; Burzyński & Śmietanka, 2009; Śmietanka, Burzyński & Wenne, 2010) and even exceeds the distance observed in *Musculista senhousia*, the only other DUI-positive member of the Mytilidae family for which both complete mitochondrial genomes are published (Passamonti et al., 2011), (Fig. 4).

The most striking feature of sex-specific mitogenomes in the cases of *Perumytilus* and *Musculista* is the presence of additional protein coding genes in both of them. However, in *M. senhousia* additional copy of *cox2* is present in the M mitogenome. It has been speculated that products of such M- specific genes may participate in the tagging of sperm-derived mitochondria thus constituting important functional element of the DUI system (Passamonti et al., 2011; Milani et al., 2013). In *P. purpuratus* the anonymous ORF is located solely in the F mitogenome. Therefore, the speculations regarding the potential function of f-ORF protein are in this case much more difficult. The residual similarity to ND4 may only indicate putative origin of this gene but the apparently very hydrophobic overall secondary structure (Fig. 4) suggests that the protein is truly buried inside a membrane —a striking difference to the f-ORF discussed in the context of *M. edulis* F mitogenomes (Breton et al., 2011a; Minoiu, Burzyński & Breton, 2016) which may only encode a relatively short polypeptide. In *P. purpuratus* this gene is under purifying selective pressure (Table 2), suggesting that its amino acid sequence is conserved by natural selection.

The relaxation of selective pressures acting on M mitogenomes was postulated soon after the discovery of DUI (Stewart et al., 1996). The expectation that M mitogenomes will always show higher genetic diversity were usually met, particularly for larger *Mytilus* population data sets (Śmietanka, Burzyński & Wenne, 2009; Śmietanka et al., 2013). However, seemingly contrasting pattern has been described in *M. senhousia* (Passamonti, 2007). In this case the population level genetic diversity was higher in the F than in the M lineage. Similar phenomenon can be observed in *P. purpuratus* (Table 2), despite the overall greater genetic distance between M than F mitogenomes from the two populations (Fig. 6). This seemingly contradictory pattern of polymorphisms can be explained by recurrent sweeps periodically resetting the polymorphism which are apparently more frequent in the M lineage. Such compensation-draft-feedback mechanism has been previously postulated for *Mytilus (Śmietanka, Burzyński & Wenne, 2010)* to explain the accelerated evolution of *atp8* and may well be responsible for this paradox.

## Conclusions

The presence of two divergent mitogenomes in *P. purpuratus* confirms that the species transmits its mitochondria to the next generation under DUI model. Only the F mitogenome contains a very long, additional ORF, possibly derived from *nd4* gene and likely involved in the functioning of DUI. The size and distribution of this ORF should make future attempts to study its function easier than in other DUI species because its fate will be relatively easy to follow with histochemical techniques. It will be easier to find good epitopes for rising specific antibodies. Future research is needed to investigate other members of Mytilidae family in mitogenomic context to see if they also show unusual DUI-related mitogenomic features.

##  Supplemental Information

10.7717/peerj.5593/supp-1 Table S1 PCR primers for *Perimytilus purpuratus* used in this study.

10.7717/peerj.5593/supp-2 Table S2 Length, base composition and sequence divergence of M and F rRNA and protein genes of *Perumytilus purpuratu* s from two mitogenomes sampled from the northern population (individual LCP1).

10.7717/peerj.5593/supp-3 Table S3 Length, base composition and sequence divergence of M and F rRNA and protein genes of *Perumytilus purpuratu* s from two mitogenomes sampled from the south-eastern population (individual BRP4).

## Additional Information and Declarations

### Data Availability

The following information was supplied regarding data availability: Annotated mitogenomic sequences have been deposited in GenBank under the following accession numbers: MH330330 (LCP1, M), MH330331 (BRP4, M), MH330332 (LCP1, F), MH330333 (BRP4, F). Raw NGS reads are available in SRA database under the following accession numbers: SRR7504346 (F amplicon) and SRR7504347 (M amplicon), SRR7504388 (RNA-Seq), SRR7504397 (genomic reads). Partial mitochondrial sequences are available under the following accession numbers: MH645667–MH645717.
