# Supplementary material for: Mitogenomics of Perumytilus purpuratus (Bivalvia: Mytilidae) and its implications for doubly uniparental inheritance of mitochondria
Source: PeerJ. 2018 Sep 18;6:e5593. doi: 10.7717/peerj.5593 (PMC6149501; doi:10.7717/peerj.5593)
Supplement: Supplementary file 1 [file peerj-06-5593-s001.odt]

Supplementary Table 1. PCR primers for Perimytilus purpuratus used in this study. Primer pairs P1 and P2 amplify F mitogenome in LR-PCR. Likewise, primer pairs P3 and P4 amplify the complete M mitogenome in LR-PCR. Primers from the “P” series are specific for the northern haplotypes. Primers from the “BS” series are universal and amplify respectively: BS1-BS2 fragment of the F mitogenome between cox3 and nd5 genes, BS3-BS4 amplify complete f-ORF from the F mitogenome and primers BS7-BS8 amplify the fragment homologous to the one amplified with primers BS1-BS2 but from the M mitogenome. Predicted melting temperatures are given, but each individual PCR must be optimized individually.

| Primer | Genome location | 5’-Sequence-3’ | Tm |
| --- | --- | --- | --- |
| P1_F | Female_nd6 | GTTATATGTGGCAGAATATTGGCGAG | 64°C |
| P1_R | Female_nd5 | CCAGCAGTCACTAGAGTAGAAGAATGAACT | 64°C |
| P2_F | Female_coIII | ATGCCTCTAAGATGTAACTCGTG | 51°C |
| P2_R | Female_cytb | CAATCTGAACAACTAAACACAACC | 51°C |
| P3_F | Male_nd6 | TGGTTTGTATAAGGCTGAGTGC | 66°C |
| P3_R | Male_nd5 | GCTGTGACCAAAGTAGAAGAGTG | 66°C |
| P4_F | Male_coIII | GGTATTCGTGCTCCAGGG | 50°C |
| P4_R | Male_cytB | CAGCAGTCAACATTCTAGTAATAACC | 50°C |
| BS1_perF | Female | AGACAAAAGCAAGACCCAGAG | 58°C |
| BS2_perF | Female | CAATTACTGGAACTTTCGCTTG | 58°C |
| BS3_perF | Female | TGCGACCCTTTATTAGAATGAAT | 58°C |
| BS4_perF | Female | GAGGTTGTTTGACTTTATGGTGAC | 58°C |
| BS7_perM | Male | GTGACTTATTACGAGAAGGTGACC | 58°C |
| BS8_perM | Male | CTAAAGCACGCATATCTTGACAG | 58°C |
