# Supplementary material for: Mitogenomics of Perumytilus purpuratus (Bivalvia: Mytilidae) and its implications for doubly uniparental inheritance of mitochondria
Source: PeerJ. 2018 Sep 18;6:e5593. doi: 10.7717/peerj.5593 (PMC6149501; doi:10.7717/peerj.5593)
Supplement: Supplementary file 2 [file peerj-06-5593-s002.odt]

Table S2 Length, base composition and sequence divergence of M and F rRNA and protein genes of Perumytilus purpuratus from two mitogenomes sampled from the northern population (individual LCP1). The number of substitutions per site from between sequences are shown, calculated according to Tamura 3-parameter model (K). The number of synonymous substitutions per synonymous site (Ks) as well as the number of nonsynonymous substitutions per nonsynonymous site (Ka) from between sequences are also shown. Standard error estimates were obtained by a bootstrap procedure (500 replicates). These analyses were conducted using the modified Nei-Gojobori (assumed transition/transversion bias = 2) model.

| Gene | M/F | Length | Base composition(%) | K | Ks | Ka |
| --- | --- | --- | --- | --- | --- | --- |
|  | T | C | A | G |
| rRNA | rrnaL | F | 1161 | 39.8 | 10.2 | 32.6 | 17.4 | 0.244 (0.018) | NA | NA |
|  | M | 1179 | 41.0 | 9.8 | 30.7 | 18.6 |  |  |  |
| rrnaS | F | 819 | 33.7 | 11.5 | 33.7 | 21.1 | 0.267 (0.025) | NA | NA |
|  | M | 819 | 34.8 | 12.0 | 33.0 | 20.7 |  |  |  |
| Protein coding genes | atp6 | F | 702 | 45.3 | 10.0 | 25.1 | 19.7 | 0.451 (0.066) | 0.661 (0.076) | 0.382 (0.041) |
|  | M | 687 | 46.6 | 11.4 | 19.7 | 22.4 |  |  |  |
| atp8 | F | 123 | 49.6 | 4.9 | 24.4 | 21.1 | 0.534 (0.244) | 0.525 (0.175) | 0.447 (0.124) |
|  | M | 123 | 51.2 | 5.7 | 13.0 | 30.1 |  |  |  |
| cox1 | F | 1587 | 41.7 | 13.8 | 23.7 | 20.9 | 0.136 (0.018) | 0.861 (0.074) | 0.106 (0.012) |
|  | M | 1611 | 40.8 | 14.3 | 22.5 | 22.3 |  |  |  |
| cox2 | F | 690 | 39.9 | 11.9 | 26.4 | 21.9 | 0.329 (0.048) | 0.772 (0.106) | 0.308 (0.036) |
|  | M | 711 | 38.1 | 13.6 | 26.0 | 22.2 |  |  |  |
| cox3 | F | 846 | 43.4 | 11.2 | 23.0 | 22.3 | 0.412 (0.052) | 0.692 (0.078) | 0.322 (0.035) |
|  | M | 804 | 45.6 | 10.9 | 19.5 | 23.9 |  |  |  |
| cob | F | 1155 | 43.5 | 13.0 | 25.9 | 17.6 | 0.088 (0.016) | 0.494 (0.048) | 0.057 (0.009) |
|  | M | 1155 | 44.1 | 12.6 | 23.6 | 19.7 |  |  |  |
| nad1 | F | 1044 | 43.4 | 10.8 | 23.5 | 22.3 | 0.285 (0.036) | 0.797 (0.089) | 0.178 (0.019) |
|  | M | 937 | 44.6 | 11.2 | 19.4 | 24.8 |  |  |  |
| nad2 | F | 984 | 43.9 | 11.7 | 22.5 | 22.0 | 0.449 (0.056) | 0.785 (0.079) | 0.318 (0.029) |
|  | M | 984 | 46.6 | 9.5 | 21.6 | 22.2 |  |  |  |
| nad3 | F | 345 | 42.9 | 11.6 | 21.4 | 21.7 | 0.393 (0.081) | 0.924 (0.171) | 0.248 (0.041) |
|  | M | 345 | 48.7 | 11.3 | 18.3 | 24.1 |  |  |  |
| nad4 | F | 1302 | 43.9 | 10.2 | 24.3 | 21.7 | 0.752 (0.085) | 0.464 (0.042) | 0.399 (0.027) |
|  | M | 1302 | 45.6 | 10.1 | 20.7 | 23.7 |  |  |  |
| nad4L | F | 270 | 41.1 | 10.4 | 23.7 | 24.8 | 0.278 (0.071) | 0.788 (0.157) | 0.251 (0.051) |
|  | M | 270 | 42.6 | 8.9 | 20.7 | 27.8 |  |  |  |
| nad5 | F | 1743 | 43.7 | 10.2 | 25.1 | 21.1 | 0.490 (0.042) | 0.707 (0.050) | 0.416 (0.027) |
|  | M | 1758 | 42.4 | 11.9 | 22.5 | 23.2 |  |  |  |
| nad6 | F | 462 | 47.7 | 8.0 | 25.8 | 20.8 | 0.585 (0.101) | 0.764 (0.103) | 00.427 (0.054) |
|  | M | 462 | 48.1 | 10.0 | 19.3 | 22.7 |  |  |  |
|  |  |  |  |  |  |  |  |  |  |  |

Note: Lengths of protein genes include the initiation codons. All stop codons were excluded from analysis.

NA, not applicable.
